# Bridging Waves and Crucial Events in the Dynamics of the Brain

**DOI:** 10.3389/fphys.2018.01174

**Published:** 2018-08-29

**Authors:** Gyanendra Bohara, Bruce J. West, Paolo Grigolini

**Affiliations:** ^1^Center for Nonlinear Science, University of North Texas, Denton, TX, United States; ^2^Information Science Directorate, Army Research Office, Durham, NC, United States

**Keywords:** brain waves, crucial events, periodicity, 1/f spectrum, coherence

## Abstract

Earlier research work on the dynamics of the brain, disclosing the existence of crucial events, is revisited for the purpose of making the action of crucial events, responsible for the 1/*f* −noise in the brain, compatible with the wave-like nature of the brain processes. We review the relevant neurophysiological literature to make clear that crucial events are generated by criticality. We also show that although criticality generates a strong deviation from the regular wave-like behavior, under the form of Rapid Transition Processes, the brain dynamics also host crucial events in regions of nearly coherent oscillations, thereby making many crucial events virtually invisible. Furthermore, the anomalous scaling generated by the crucial events can be established with high accuracy by means of direct analysis of raw data, suggested by a theoretical perspective not requiring the crucial events to yield a visible physical effect. The latter follows from the fact that periodicity, waves and crucial events are the consequences of a spontaneous process of self-organization. We obtain three main results: (a) the important role of crucial events is confirmed and established with greater accuracy than previously; (b) we demonstrate the theoretical tools necessary to understand the joint action of crucial events and periodicity; (c) we argue that the results of this paper can be used to shed light on the nature of this important process of self-organization, thereby contributing to the understanding of cognition.

## 1. Introduction

Following the dynamics of the brain is a challenging issue that has forced researchers to go beyond applying the conventional forms of non-equilibrium statistical physics (Papo, 2013) and is expected to contribute to reshaping the emerging field of complex networks as well (Papo et al., 2014). The dynamics of the brain and of biological processes in general, are characterized by homeodynamics (Yates, 1994), thereby implying that the analysis of biological dynamics ought to be done taking into account that it is typically driven by rhythms and waves.

A parallel line of inquiry has recently been developed that focuses on the connection between the dynamics of the brain and the phenomenon of criticality (Aburn et al., 2012; Tagliazucchi et al., 2012; Boonstra et al., 2013). Criticality in the brain is a subject widely discussed in the neurophysiology literature, as reviewed in the recent paper (Cocchi et al., 2017) and is a term adopted by physicists to denote, for instance, the spontaneous magnetization of a material below the Curie temperature. At the critical temperature *T*_*C*_ where this transition occurs, a long-range correlation between the thermally disordered spins occurs. It is widely thought that an analogous condition is fulfilled by brain dynamics with the consequence of strongly correlating the functionality of different physical regions of the brain. This connection between brain dynamics and phase transition processes at criticality led the present investigators to focus on the concept of crucial events.

For context, let us briefly consider how the concept of crucial events was introduced in neurophysiology. Contoyiannis and Diakonos (Contoyiannis and Diakonos, 2000) studied a 3-dimensional Ising model at criticality and proved it to be the generator of Type I intermittency. In the sequel (Contoyiannis et al., 2002) they pointed out that this connection with Type I intermittency can be formally expressed through a waiting-time probability density function (PDF) ψ(τ), with a dominant inverse power law (IPL) structure, with IPL index μ. This PDF is exponentially truncated at long times. Using the intermittency language (Manneville and Pomeau, 1979; Pomeau and Manneville, 1980) a laminar region is interpreted as a quiet zone between short regimes of rapid erratic activity. The durations of different laminar regions are uncorrelated and the turbulent processes separating consecutive laminar regions are the crucial events studied in this paper. The discovery of crucial events in the field of turbulence (Manneville and Pomeau, 1979; Pomeau and Manneville, 1980) had deep consequences in statistical physics. In fact, if the IPL index μ of ψ(τ) is smaller than 3, the process hosting the crucial events is not ergodic. On the other hand, the statistical analysis of molecular diffusion in biological cells (Metzler et al., 2014) shows that these processes are not ergodic, since they host crucial events. This leads to the inequality μ < 3 as being an important condition for the definition of crucial events. It has to be stressed that ergodicity breakdown seems to be a general property of biological processes (Grigolini, 2015), not limited to the specific case of molecular diffusion in biological cells.

Returning to neurophysiology, we can make the conjecture that crucial events in the EEGs are signaled by abrupt transitions from regular to a fast irregular behavior, Rapid Transition Processes (RTPs). Allegrini et al. (2010) searched for these events in the brain of healthy patients using a technique for identification of RTPs in the brain activity proposed by brothers Fingelkurts and co-workers (Fingelkurts, 1998; Fingelkurts and Fingelkurts, 2005, 2006, 2008, 2015; Kaplan et al., 2005). After detecting them, Allegrini et al. (2010) proved that, as expected, the RTPs host crucial events.

On the basis of earlier remarks, crucial events are defined in terms of their statistical properties as follows. The time intervals between consecutive crucial events are described by the waiting-time PDF ψ(τ) having an IPL structure

(1) $$\begin{array}{r} \psi \left(\tau \right)\propto \frac{1}{\tau^{\mu }}, \end{array}$$

with the IPL index μ in the interval

(2) $$\begin{array}{r} 1<\mu <3. \end{array}$$

From the earlier arguments, furthermore, it is clear that the crucial events are renewal and consequently the times τ_*i*_ should not be correlated. If a sequence of crucial events are defined by the time intervals τ_1_,τ_2_,τ_3_,… then the time-average correlation function is a Kronecker delta function where the time average is indicated by an overbar

(3) $$\begin{array}{r} C\left(t\right)=\frac{\underset{|i-j|=t}{\sum }\bar{\left(\tau_{i}-\bar{\tau }\right)\left(\tau_{j}-\bar{\tau }\right)}}{\underset{i}{\sum }\bar{{\left(\tau_{i}-\bar{\tau }\right)}^{2}}}. \end{array}$$

This correlation function is properly normalized, thereby yielding *C*(0) = 1, and in the case of genuine renewal events should satisfy the condition *C*(*t*) = 0 for *t* > 0. This renewal property can also be expressed by the assumption that the probability of occurrence of both τ_*i*_ and τ_*j*_, Π(τ_*i*_, τ_*j*_), when *i* ≠ *j* is given by,

(4) $$\begin{array}{r} \Pi \left(\tau_{i},\tau_{j}\right)=P\left(\tau_{i}\right)P\left(\tau_{j}\right), \end{array}$$

where *P*(τ_*i*_) and *P*(τ_*j*_) are the probability of occurrence of τ_*i*_ and τ_*j*_, respectively.

Allegrini et al. (2010) following the line of thoughts illustrated in this Introduction conjectured that these crucial events are a signature of criticality and addressed the important tasks of detecting them from the observation of EEG time series (Coles and Rugg, 1995). The criticality hypothesis is in line with the views of many other researchers (Levina et al., 2007, 2009; Haimovici et al., 2013). In conclusion, the widely shared idea that the brain operates at criticality led to the discovery that crucial events act on the brain. This important conclusion left open two important problems, hereby illustrated. Their solutions are the main results of this paper.

### 1.1. First problem

It is not yet clear what kind of criticality generates crucial events, either that determined by externally tuning a control parameter (Ising-like), or that achieved spontaneously through the internal system dynamics, i.e., self-organized criticality (SOC), is expected to afford a sufficient theoretical picture. We discuss the open issue of the proper form of criticality to use to increase our understanding of the brain dynamics in sections 5 and 6.

Here we stress that the research lines of this paper are determined by the recent form of self-organization called self-organized temporal criticality (SOTC) (Mahmoodi et al., 2017, 2018a,b; Mahmoodi et al., to be submitted). This form of criticality has been proposed for the purpose of establishing a new view of the field of Evolutionary Game Theory. The authors of this field explain the emergence of cooperation, in spite of the incentive for the single individual to cheat, and are looking for the spontaneous emergence of the global cognition that cooperation generates more social benefits, and consequently more benefits for the single individuals. SOTC shows that this cognition emerges naturally from a social interaction where the degree of social attention, which is related in some way to the control parameter of the ordinary approaches to phase transitions, is changed by the single individuals and it increases or decreases according to whether their overall social benefit increases or decreases.

### 1.2. Second problem

The second problem left unsettled by the results of Allegrini et al. (2010) is how to make crucial events compatible with homeodynamics (Yates, 1994). Although SOTC has a sociological origin, it can also be applied to neurophysiological processes and more generally to biological processes satisfying the principle of homeodynamics. In this case the single units are oscillators that adjust their interactions with other units so as to maximize the reciprocal synchronization (Mahmoodi et al., to be submitted). This extension of SOTC yielding the emergence of global periodicity makes it difficult to make analytical predictions. Recent work (Lambert et al., to be submitted) bypasses this difficulty: it is a promising way to take homeodynamics into account so as to make periodic behavior compatible with crucial events, replacing extensive computations with analytical equations. This theoretical approach establishes a connection between coherence and criticality-induced crucial events, and was recently used (Tuladhar et al., 2018) to evaluate the influence of meditation on brain dynamics (Tuladhar et al., 2018) by means of the statistical analysis of the heart rate variability time series of these subjects.

The main result of the present paper is establishing an approach that simultaneously detects the statistical properties of crucial events and a connection with the wave-like nature of brain dynamics. The adoption of the RTP method is very attractive but, as shown in section 2, its adoption does not make it possible for us to measure the complexity of brain dynamics directly and in addition requires a filtering process. Herein we propose a technique of analysis of EEG time series data not requiring the detection of RTPs, and that leads to the detection of scaling directly from raw data. We show that the resulting scaling is identical to that obtained in earlier work using RTP's. More importantly, the present technique helps establish a bridge between EEG waves (di Santo et al., 2018) and crucial events. In fact, as shown in section 3, this bridge is expected to lead us to understand more about what form of criticality to apply to study the dynamics of the brain.

### 1.3. Outline of the paper

In section 2 we review the procedure adopted to detect RTP events. We devote section 3 to an intuitive introduction to the process of self-organization combining periodicity and crucial events and in section 4 we analyze the spectrum of one EEG to point out the interesting qualitative agreement with the predictions of section 3 . In section 5 we illustrate a technique of detection of crucial events that facilitates the analysis of EEG time series. Finally, in section 6 we draw some conclusions and present plans for future work.

## 2. Detection of rapid transition events

As mentioned earlier, the efficacy of the RTP method in the study of brain dynamics has been established by the brothers Fingelkurts and co-workers (Fingelkurts, 1998; Fingelkurts and Fingelkurts, 2005, 2006, 2008, 2015; Kaplan et al., 2005). The connection between RTPs and crucial events has been demonstrated by Allegrini et al. (2010). However, for the sake of clarity we sketch here the approach adopted by the latter authors to obtain crucial events from RTPs, to emphasize the importance of recovering the same results for detecting the same scaling, using a very different technique. We base our analysis on data derived from Fingelkurts (1998), which are available in physionet.org Fingelkurts and Fingelkurts (2005).

**Figure 1:**
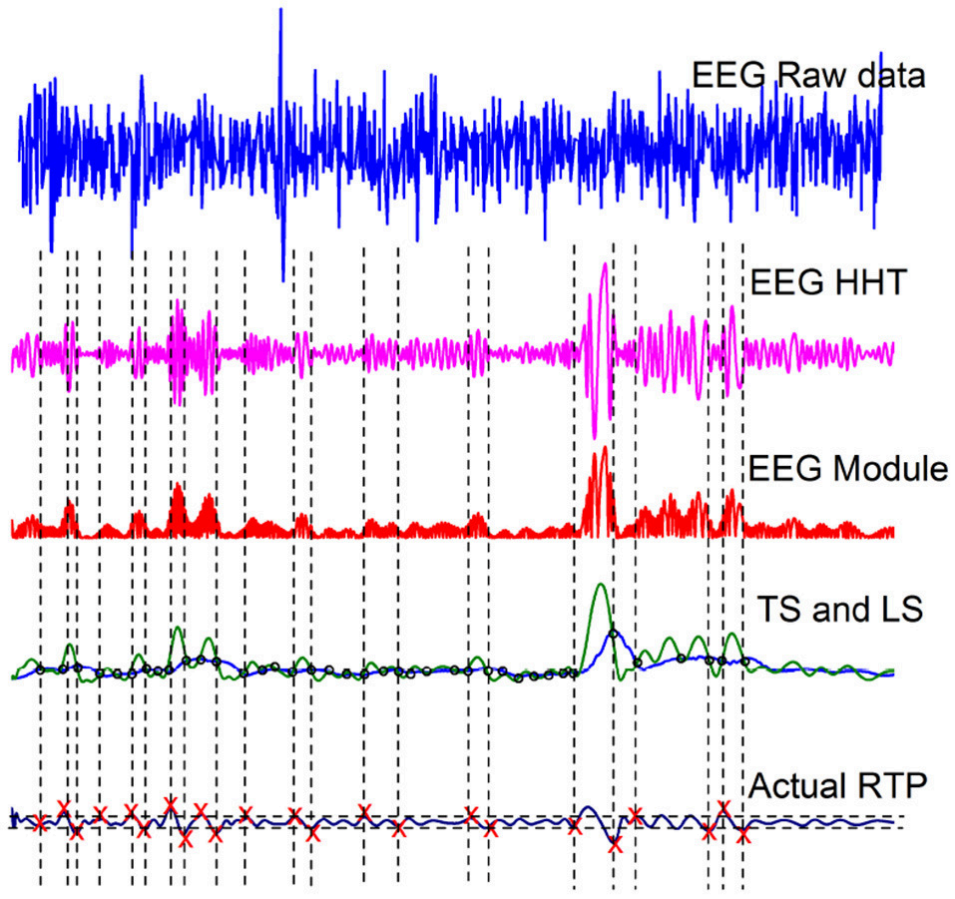
Illustration of the RTP procedure of Kaplan et al. (2005).

These EEG time series data have been filtered between 0.15-28 Hz and the sampling rate (*F*_*s*_) is 2048 Hz. We select one healthy subject, from the dataset, and the top panel of Figure 1 shows the raw data of this subject. The second panel displays one intrinsic mode function, obtained adopting the method of Hilbert-Huang Transformation (HHT) illustrated in Huang and Wu (2008). The HHT method decomposes the original EEG signal into many intrinsic mode functions, with different structures. We select a structure with a kind of sausage-like pattern suggested by the theoretical results of (Bologna et al., 2010). They proved that a set of infinitely many three-state oscillators, cooperatively interacting with an interaction parameter *K*, at criticality generate a coherent non-harmonic oscillation. When the number of oscillators is finite the regular non-harmonic oscillations generate a sausage-like structure similar to the second panel of Figure 1. In section 6.1 we make additional remarks on the criticality-induced sausage-like structure.

The third panel from the top of Figure 1 is the modulus of the data depicted in the second panel. The green curve in the fourth panel is the envelope of the curve of the third panel, called a Testing Sequence (TS). The blue curve in the fourth panel is the Level Sequence (LS) obtained from the TS by means of a running-average smoothing. Finally, the red crosses in panel five denote crucial events.

Note that this procedure for finding the crucial events is not sufficiently accurate to be restricted to detecting only renewal events. It is known that the events revealed by this analysis are a mixture of crucial events and ordinary Poisson events (Allegrini et al., 2010). However, the presence of Poisson events does not prevent us from detecting the anomalous scaling generated by the crucial events. The desired scaling is detected in the following way. The authors of Grigolini et al. (2001) use the detected events to generate a diffusion process *x*(*t*) by means of the rule that the random walker jumps ahead when an event, either crucial or Poisson, occurs. The scaling generated by the Poisson events has a power-law index δ = 0.5, whereas the scaling power-law index δ of the crucial events is given by the important relation

(5) $$\begin{array}{r} \delta =\frac{1}{\mu -1}. \end{array}$$

Note that the latter scaling dominates asymptotically in the time due to Equation (5) resulting in δ > 0.5 when the condition 2 < μ < 3 applies (Grigolini et al., 2001). When 1 < μ < 2 crucial events yield the scaling δ = (μ−1), but the EEG time series studied in this paper and the subordination theory of section 3, adopted to explain their complexity, show that we need to focus on μ > 2.

To be explicit, since in this paper as far as the scaling detection is concerned, we adopt the same procedure as that proposed by Grigolini et al. (2001), we generate a fluctuation ξ(*t*) holding the value 1 when an event, either crucial or Poisson, occurs, and the vanishing value when no event occurs. The diffusion variable *x*(*t*) is obtained from the following equation of motion

(6) $$\begin{array}{r} \frac{d}{dt}x=\xi \left(t\right). \end{array}$$

Using a moving window of size *t*, we generate a PDF *p*(*x, t*) and the Shannon information entropy

(7) $$\begin{array}{r} S\left(t\right)=-\int_{-\infty }^{+\infty }dxp\left(x,t\right)ln\left[p\left(x,t\right)\right]. \end{array}$$

The PDF constructed from the diffusion process has the scaling form

(8) $$\begin{array}{r} p\left(x,t\right)=\frac{1}{t^{\delta }}F\left(\frac{x}{t^{\delta }}\right). \end{array}$$

Then inserting Equation (8) into Equation (7), after some algebra yields

(9) $$\begin{array}{r} S\left(t\right)=A+\delta ln\left(t\right), \end{array}$$

where *A* is the entropy constant

(10) $$\begin{array}{r} A\equiv -\int_{-\infty }^{+\infty }dyF\left(y\right)ln\left[F\left(y\right)\right]. \end{array}$$

To make this treatment compatible with the subsequently discussed arguments about intermediate asymptotics, we rewrite Equation (9) in the following way

(11) $$\begin{array}{r} S\left(t\right)=C+\delta \left(t\right)ln\left(t\right), \end{array}$$

where *C* denotes a constant that may differ from *A*, when, as we find herein, the proper complexity scaling emerges only in the region of *intermediate asymptotics*.

**Figure 2:**
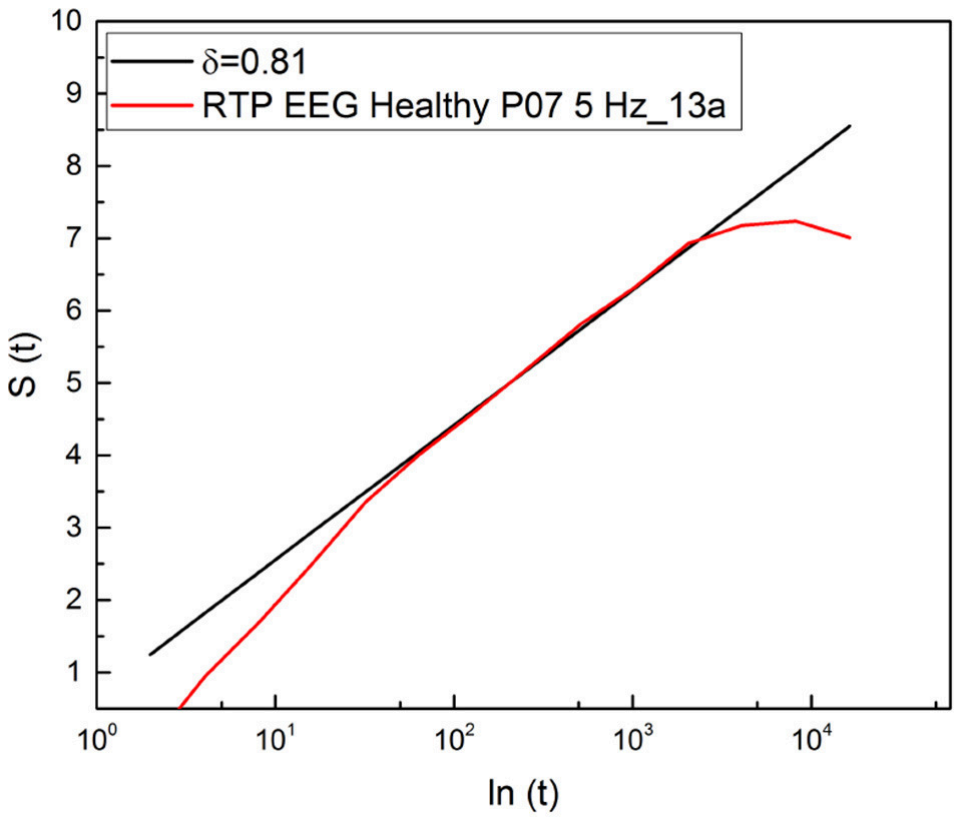
Detection of the scaling δ applying DEA to the diffusion process generated by a random walker making a jump ahead when a crucial event occurs.

It is important to stress that a significant advance of the theoretical justification of Equation (11) based on an extension of the theory of SOC, incorporating complexity in the time domain, is called SOTC (Mahmoodi et al., 2017, 2018a,b). This new theory provides a rationale for the crucial IPL index μ. In fact, according to SOTC the processes of spontaneous self-organization, in general, and especially those behind the statistical analysis used herein, namely physiological processes, naturally evolves to a state generating the crucial events defined in section 1. These events manifest scaling in the intermediate time domain, called intermediate asymptotics (Goldenfeld, 1992; Barenblatt, 1996). As a consequence of temporal complexity emerging in the intermediate time scale, *S*(*t*) is not a straight line when expressed as a function of *ln*(*t*). As shown in Figure 2, entropy is a straight line in the intermediate time region and its slope is used to define the statistics of crucial events occurring within that region through the IPL index μ, established by Equation (5). This data analysis technique has been labeled the diffusion entropy approach (DEA).

To explain using an intuitive interpretation the intermediate asymptotics, we notice that the short-time region corresponds to the time scale where the self-organization is not yet perceived by the interacting units. According to SOTC (Mahmoodi et al., 2017), the intermediate time scale with temporal complexity becomes more and more extended as the number of units cooperatively interacting increases. However, the fluctuation intensity becomes smaller and the long-time scale is a sort of Poisson shoulder that, however, does not affect the communication efficiency of the complex system, since that efficiency is determined by the intermediate time region. The exponential truncation favors the transmission of information, because the flexibility of the complex system's response to the environment requires that the system explore a sufficiently high number of crucial events (*free-will states*) to adapt itself to the external influence. It has the effect of making the mean value of the time interval between consecutive crucial events finite, even in the case μ < 2. We remind the reader that the theoretical mean time between crucial events is < τ > ∝1/(μ−2), if μ > 2 and it is divergent if μ < 2 and the IPL PDF is not truncated.

The events generated by SOTC are renewal, which explains adopting Equation (5) for the connection between δ and μ, which is based in fact on the renewal assumption (Grigolini et al., 2001). We see from Figure 2 that the subject examined with DEA procedure yields μ = 2.2.

It is convenient to stress the fact that the choice of the RTP method, illustrated in Figure 1, has been motivated by the intuitive assumption that crucial events have physical effects. With reference to the second panel from the top of Figure 1, an event may be located in the short-time region of weak fluctuations separating the ending of one sausage from the beginning of another. Actually, the theoretical approach outlined in section 3 suggests that many more crucial events exist, thereby leading to a scaling method evaluation resting on a much larger number of crucial events, even if, as in the case of the earlier mentioned method by the brothers Fingelkurts and co-workers (Fingelkurts, 1998; Fingelkurts and Fingelkurts, 2005, 2006, 2008, 2015; Kaplan et al., 2005), not all the events adopted to generate the diffusion process analyzed with DEA, are crucial.

## 3. Subordination

Establishing the statistics of the crucial events manifest in EEG time series by means of the detection of RTP, unfortunately, does not help us to build a bridge between the wavelike nature of EEG time series and crucial events. To establish the theoretical connection between the periodicity of EEG time series and crucial events, we adopt the SOTC model of units with an individual periodicity, for instance the SOTC model (Mahmoodi et al., 2017) as applied to the Kuramoto model (Kuramoto, 1975). This computationally demanding approach, however, has not yet been converted into an analytical approach for bridging the gap between waves and crucial events. We believe that the theoretical remarks of this section are a fair account of this form of SOTC.

The research work done in the recent past on the brain with the help of the RTP method led Allegrini et al. (2009) to conclude that the crucial events are characterized by values of μ very close to μ = 2, according to the prescription

(12) $$\begin{array}{r} S\left(\omega \right)\propto \frac{1}{\omega^{3-\mu }}. \end{array}$$

The derivation of this spectrum was done by other researchers (Margolin and Barkai, 2006; Lukovic and Grigolini, 2008), who assumed that the time regions between consecutive crucial events are filled with either +1′*s* or −1′*s*, values generated by a coin tossing algorithm. However, no direct evaluation of the EEG spectrum was done. Herein we see that if the EEG spectrum is evaluated, the frequency region for ω → 0 is affected by strong fluctuations making it difficult to assess the IPL property of Equation (12). However, we are able to shed light into the overall structure of the spectrum and we argue that this is compatible with Equation (12).

To establish a bridge between crucial events and periodicity, as done by Ascolani et al. (2009), we make an extension of the well known Continuous Time Random Walk (CTRW) (Montroll and Weiss, 1965; Sokolov, 2000; Shlesinger, 2017). The subordination to a coherent process with frequency Ω is a mathematically simple way of simulating a genuine process of self-organization. We have a clock, the hands of which move clockwise with frequency Ω from noon to noon, making *T*_*RR*_ clicks with the time interval Δt between one click and the next. Thus,

(13) $$\begin{array}{r} \Omega =\frac{2\pi }{T_{RR}\Delta t}. \end{array}$$

The crucial events, some of which have been detected by Allegrini et al. (2010) through the search of RTP, are imbedded into this regular motion, by assuming that the time interval between consecutive clicks is derived from a waiting-time PDF ψ(τ) with the temporal complexity of Equation (1). The explicit analytical form of ψ(τ) is

(14) $$\begin{array}{r} \psi \left(\tau \right)=\left(\mu -1\right)\frac{T^{\mu -1}}{{\left(\tau +T\right)}^{\mu }}, \end{array}$$

corresponding to the survival probability

(15) $$\begin{array}{r} \text{Ψ}\left(\tau \right)={\left(\frac{T}{\tau +T}\right)}^{\mu -1}. \end{array}$$

The parameter *T* serves the purpose of properly defining the short-time scale and setting the normalization condition Ψ(0) = 1. The temporal complexity becomes important at times τ ≫ *T*.

This procedure of infusing the original perfect coherence of the clock with complex randomness establishes a bridge between waves and crucial events. This has the effect of turning the frequency Ω into an effective frequency Ω_*eff*_, thereby modeling a process of self-organization of interacting oscillators, each of which is characterized by its own frequency, into a collective homeodynamic process.

According to the theoretical treatment of Lambert et al. (to be submitted), the effective frequency is, valid for μ > 2,

(16) $$\begin{array}{r} \Omega_{eff}=\frac{\Omega \left(\mu -2\right)}{T}. \end{array}$$

**Figure 3:**
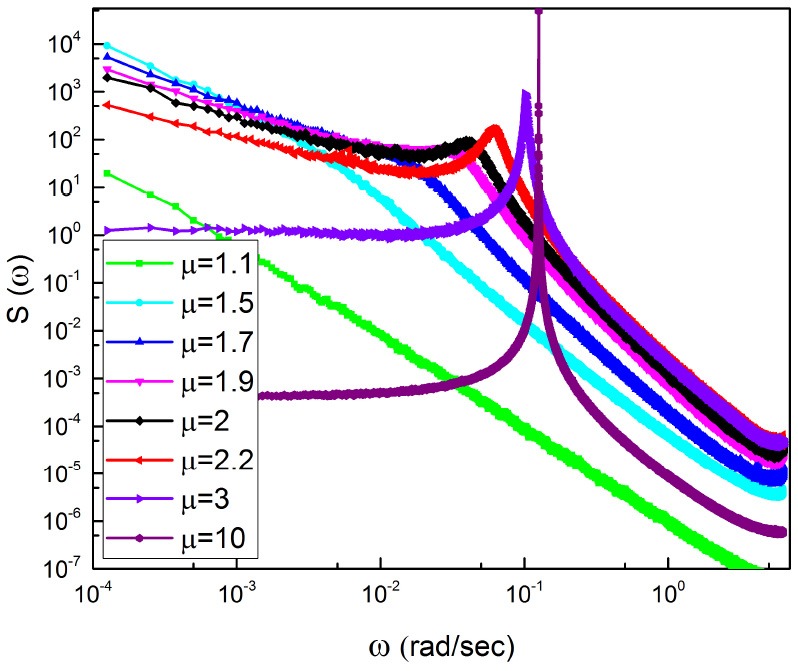
Power Spectra obtained averaging over 300 trajectories with numerical parameters *T* = 0.5 and the regular oscillation before subordination has the frequency Ω = 0.77. The inlay denotes the calculations for IPL indices spanning a range from 1.1 to 10.

This theoretical prediction suggests, in agreement with Figure 3, that the frequency peak is evident for μ > 2 and that, in addition, it also depends on the parameter *T* of the waiting-time PDF ψ(τ) of Equation (14). This property is used in section 4 to shed light into the meaning of the HHT components of the method adopted to detect RTP events Huang and Wu (2008). In the range μ > 2, when both the first and second moment of τ are finite, Ω_*eff*_ = Ω (Lambert et al., to be submitted).

This illustration of subordination makes it evident that crucial events are not only at the border between consecutive pieces of the sausage but the oscillatory-like behavior within a sausage hosts crucial events. It is surprising that the same conclusion has been achieved by the Fingelkurts brothers (Fingelkurts and Fingelkurts, 2005) who wrote “By varying the parameters of this technique it is possible to obtain the segments corresponding to a more or less detailed structure of the EEG/EMG. Therefore, there are prospects for the description of the structural EEG/EMG organization as a hierarchy of segmental descriptions on different time-scales. We make the conjecture that this may have the effect of establishing a correlation between the non-crucial events, thereby contributing a significant theoretical advance on the issue of cognition, and the effects of meditation on the dynamics of the brain (Tuladhar et al., 2018). This observation suggests that it should be possible to design a method of statistical analysis for extracting information from a larger set of crucial events even if they remain invisible.

Figure 3 illustrates spectra generated by surrogate sequences obtained using the subordination method with Δ*t* = 1. We keep the frequency Ω fixed and change the IPL index μ. We note that a spectrum consists of three parts. There exists a peak corresponding to the effective frequency Ω_*eff*_ that shifts to the right upon decreasing μ and disappears for μ < 2. At the left of the Ω_*eff*_ peak the slope of the spectrum β is determined to be

(17) $$\begin{array}{r} \beta =3-\mu . \end{array}$$

We see that the spectrum becomes flat at μ = 3 and remains flat for higher values of μ, as clearly shown in Figure 3.

Note that due to the average of many realizations, which is not possible with real EEG time series, the region of low frequency is regular and is not affected by the fluctuations that would appear when evaluating the spectrum with only one time series. For this reason, the adoption of surrogate time series makes it possible for us to prove that, as expected, subordination is compatible with the emergence of 1/*f* −noise in the ideal case μ = 2.

**Figure 4:**
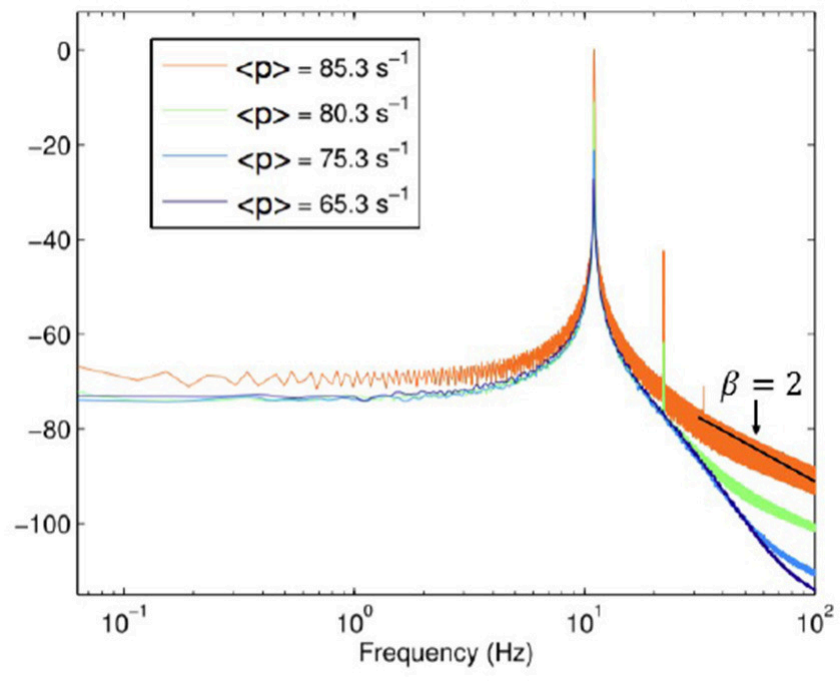
Power Spectra derived with permission from Aburn et al. (2012) under the terms of the Creative Commons Attribution License http://creativecommons.org/licenses/by/3.0/.<p>denotestheneuronfiringrate.

Let us now discuss the spectra depicted in Figure 4, which was obtained by Aburn et al. (2012) by approaching the Hopf supercritical bifurcation in order to better understanding the alpha rhythm of human EEG time series. We note that this spectrum is similar to those of Figure 3 when μ ≥ 3. In other words, this theory does not involve crucial events. It is interesting to notice that for values of ω larger than the peak frequency, the spectrum depicted in Figure 4 yields the slope β = 2. The comparison between Figures 3, 4 indicates that subordination creates a bridge between periodicity and crucial events, while making the high-frequency region identical to that of a spectrum with no crucial events.

When μ < 3 there exists a close connection between periodicity and complexity, as indicated in Figure 3 showing that the position of the periodicity bump is very sensitive to the temporal complexity.

## 4. Spectra from raw data

**Figure 5:**
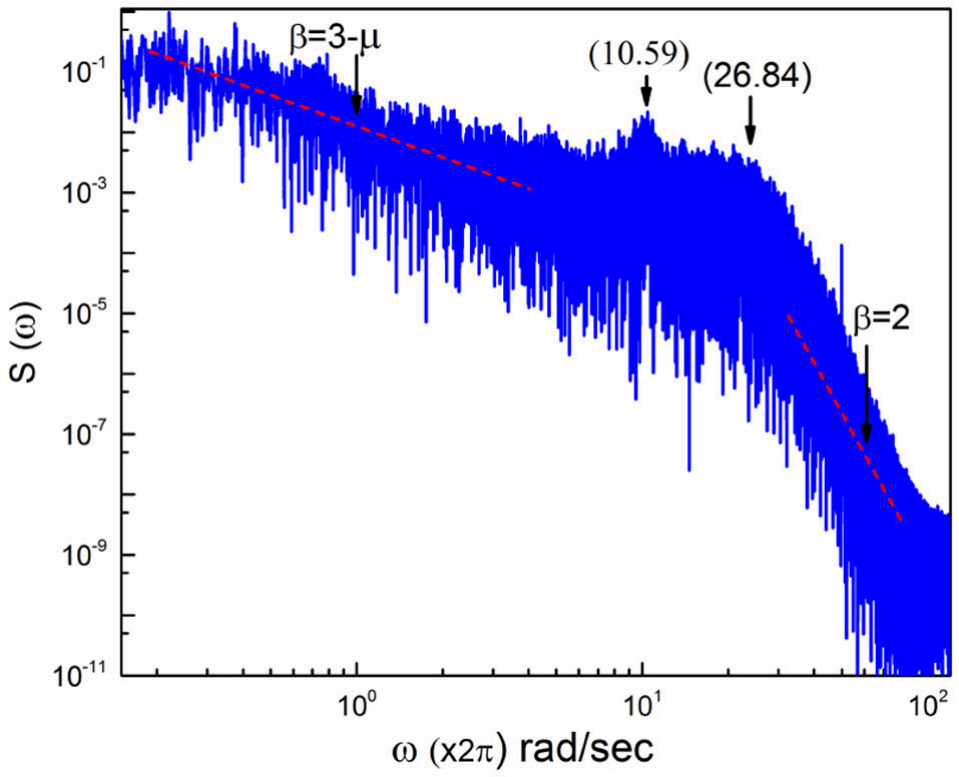
Power Spectrum obtained from raw EEG time series data.

In this section we discuss the spectrum generated by real EEG time series fluctuations as shown in Figure 5. We see that the region of low frequencies is very erratic, due to the fact that, as mentioned earlier, the use of only one time series makes it impossible to generate a smooth curve. There exists an indication of a frequency bump, generated by periodicity, and for frequencies larger than this bump the slope β = 2 is rediscovered. This real spectrum depends on a wide swath of frequencies.

**Figure 6:**
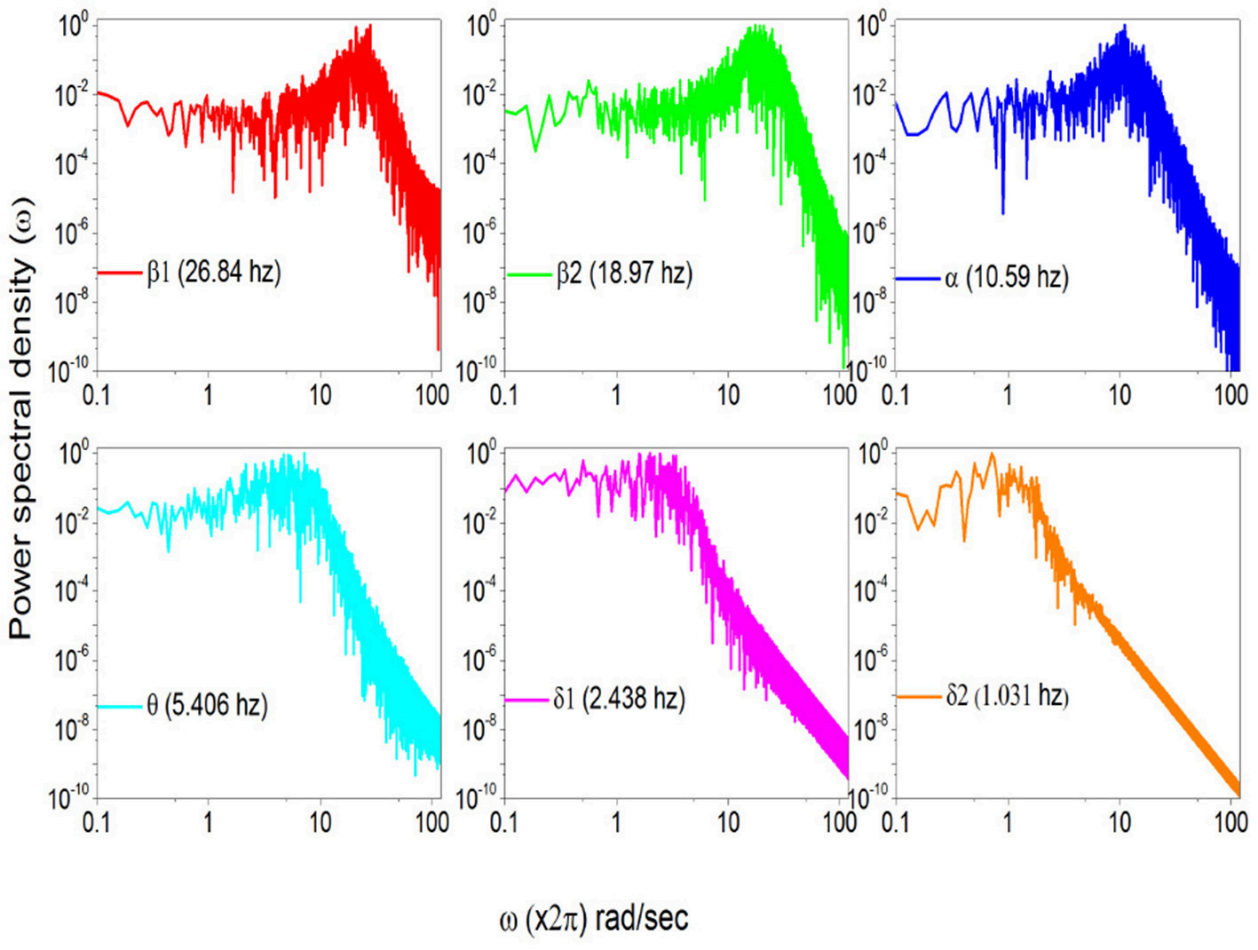
Power Spectra of the same subject with different HHT frequency components.

To stress the multi-frequency nature of the real spectrum, again using the HHT method (Huang and Wu, 2008) we evaluate six intrinsic mode functions of the EEG raw data corresponding to the spectrum of Figure 5. These different components of the whole signal correspond to six different frequencies of decreasing value. They are the frequencies: 26.84; 18.97 hz, 10.59 hz; 5.406 hz and 2.438 hz and 1.031 hz, which are shown in Figure 6.

**Figure 7:**
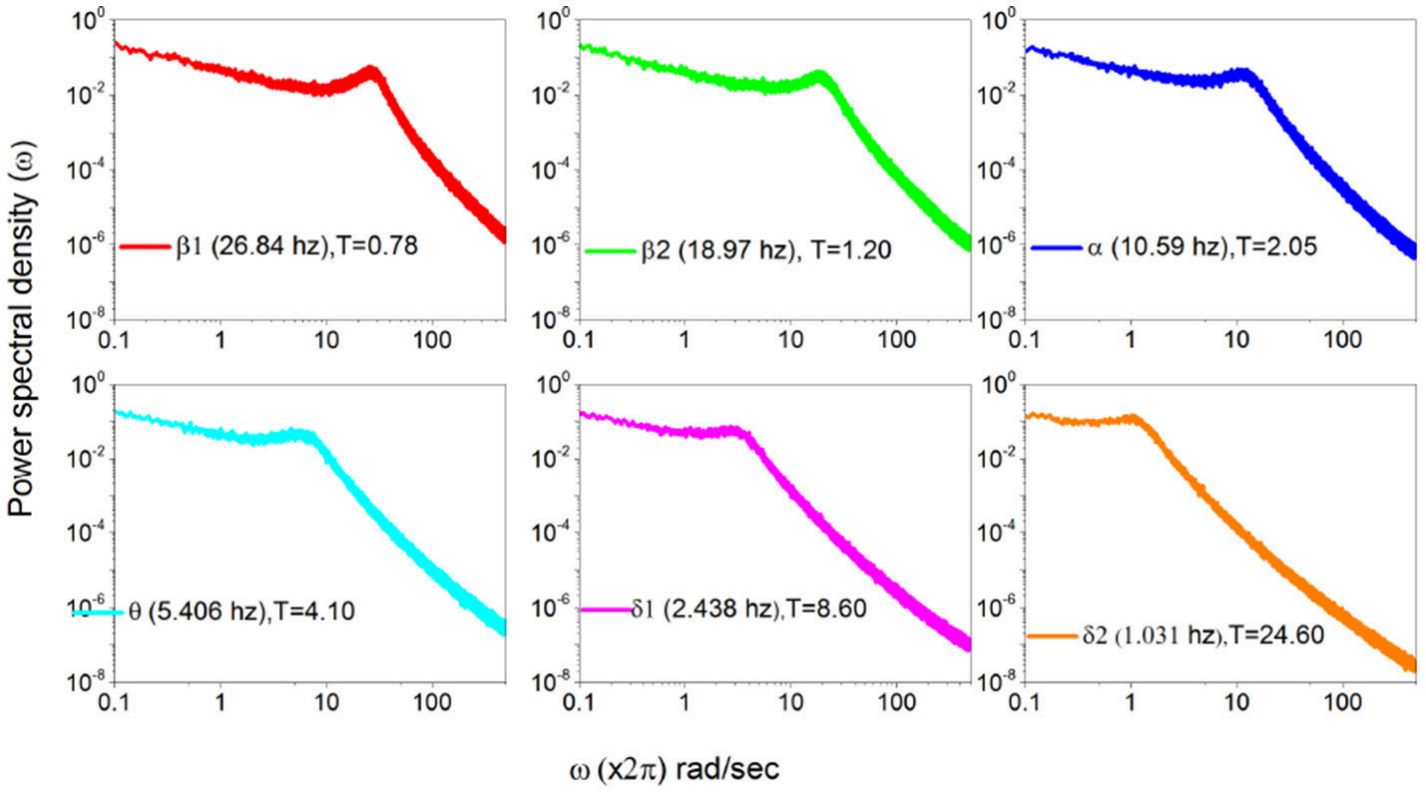
Power Spectra generated using the data recorded in Figure 6 according to subordination of section 3.

In Figure 7 we use the subordination prescription described in section 3, with Δ*t* given by an inverse sampling frequency of real data ($\Delta t=\frac{1}{2048}sec$) to generate surrogate spectra, helping to clarify the meaning of the different spectra in Figure 6. We assign to the monochromatic frequency 62 hz with six different values of the parameters *T*, mimicking the dominant frequencies of the six HHT components illustrated in Figure 6. The real spectrum of Figure 5 is interpreted as a superposition of the spectra illustrated in Figure 7. In fact all these spectra share the property β = 3−μ in the low frequency region and the property β = 2 in the high frequency regions and intermediate region where the change of slope occurs is significantly broader than in the monochromatic case.

## 5. Method of stripes

The stripe method was originally adopted to detect the scaling of crucial events hosted by heartbeats (Allegrini et al., 2002) and was not used in the case of EEG time series in all likelihood because of the lack of a proper theoretical understanding of the connection between crucial events and periodicity. The same method was more recently applied by Bohara et al. (2017) to establish a connection between the occurrence of crucial events and multifractality.

In section 3 we used an intuitive illustration of the process of self-organization, based on subordination that affords theoretical support for the adoption of the method of stripes. The central idea is that the RTP method detects only a small fraction of crucial events, whereas real EEG time series and subordination theory with them, host a much larger number of crucial events, even if they remain invisible.

**Figure 8:**
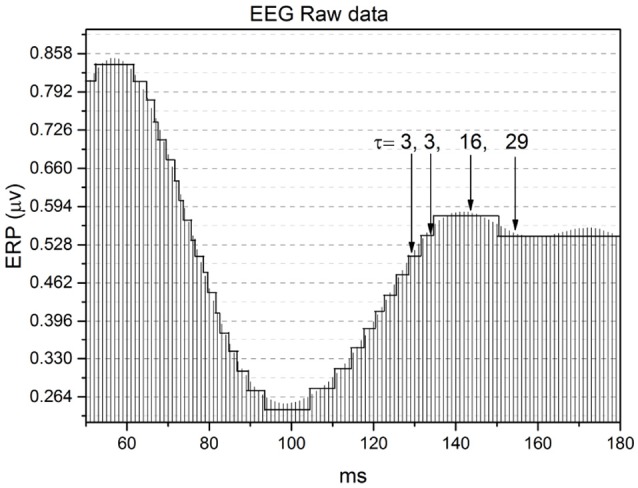
Illustration of the method of the stripes. The size of stripes is Δ*E* = 1/30 μv.

Figure 8 shows how the method of stripes works. As is well known (Coles and Rugg, 1995), an EEG time series captures Event Related Potentials (ERPs) which, in turn, measures the rate of firing neurons. The method divides the vertical axis into many stripes of size Δ*E*, here assumed to have the value Δ*E* = 1/30 μv, and we record the times at which the raw signal crosses the line separating two adjacent stripes. The level of the stripe is determined by the number of neuron firings at a given time, and we record for how long that firing rate remains constant.

The change from one firing rate to another is an event. Of course this event is not necessarily a crucial event. As a consequence, the time interval between consecutive events cannot be used to define the important parameter μ. This lack of precision in determining the occurrence of crucial events applies also to the RTP method. Let us call *N*_*T*_ the total number of events detected, *N*_*c*_ the total number of (unknown) crucial events and *N*_*nc*_ the total number of non-crucial, possibly Poisson events. The intermediate asymptotics, revealing the complex scaling δ of Equation (5), begins earlier upon increase of the ratio

(18) $$\begin{array}{r} r_{c}\equiv \frac{N_{c}}{N_{T}}. \end{array}$$

In both cases, the adoption of the DEA method is essential. In fact, after recording events with the method of stripes, as done with the method of RTP, we adopt the prescription of Grigolini et al. (2001). We again turn the sequence of detected events, either crucial or not, into a diffusion signal *x*(*t*) by making the random walker jump ahead by a fixed quantity, equal to 1. As pointed out in section 2, the non-crucial events generates a diffusion process with scaling δ = 0.5 and the crucial events, on the contrary, generate the scaling index of Equation (5) that for μ > 2 is larger than 1/2, thereby making it possible for DEA to establish the correct scaling of Equation (5) at long times.

**Figure 9:**
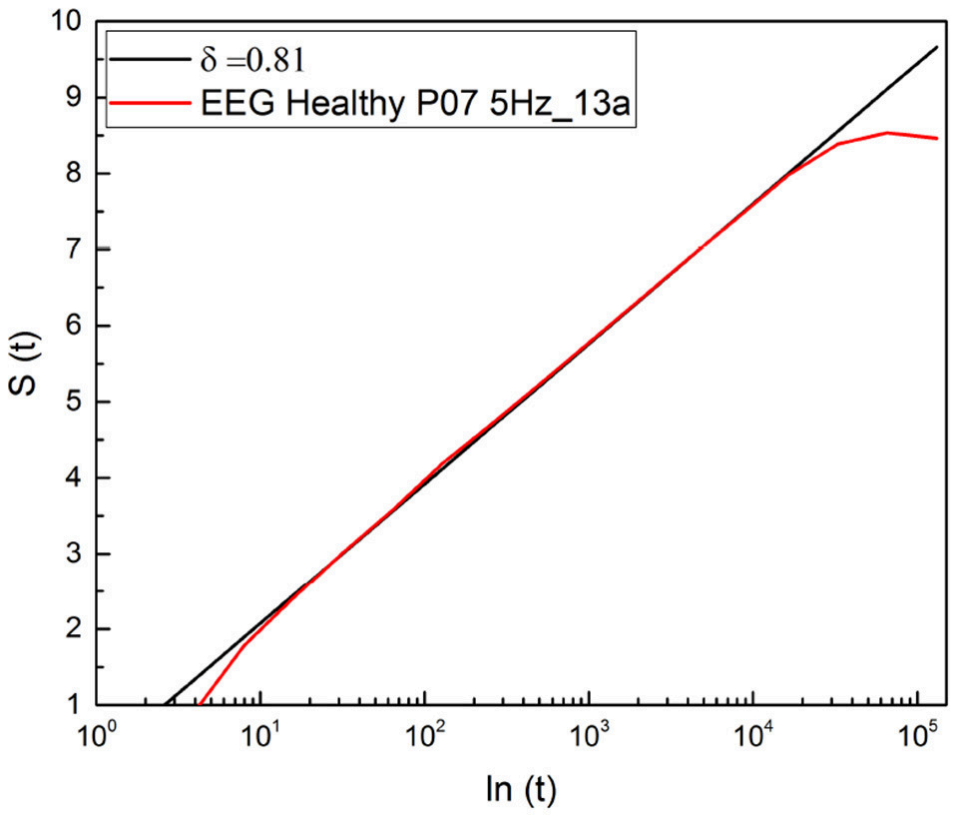
DEA applied to the diffusion process generated by the stripe-crossing events.

The result illustrated in Figure 9 shows that the scaling detected with this method is virtually identical to the result obtained with the use of RTP method. In both cases the complex scaling of Equation (5) appears in the intermediate time regime, but it appears that the present method is more accurate, since the slope covers three decades of scale, see Figure 9, while the RTP method is limited to only two decades (Figure 2). This is a clear indication that the method of stripes makes the ratio *r*_*c*_ of Equation (18) significantly larger than the RTP method.

## 6. Concluding remarks

The adoption of the RTP method makes it easy to establish the non-local nature of the brain criticality (Allegrini et al., 2010). This is a consequence of the fact that it is sufficient to count how many electrodes undergo RTP's at the same times. However, the detailed illustration in section 2 shows that the procedure to establish the occurrence of an RTP is not straightforward and we conjecture that it may be replaced by the adoption of a cross-correlation function between the EEG time series generated by different electrodes. But this remains to be verified.

### 6.1. Self-organized temporal criticality(SOTC)

The sausage-like structure of the model studied by Bologna et al. (2010) has a surprising similarity to the second panel from the top of Figure 1. This is not accidental. In fact, the theory adopted by (Bologna et al., 2010) is a phase transition obtained by the control parameter *K* taking on the critical value *K* = 1.5. At criticality the oscillators are no longer independent of one another and as a result of highly correlated motion they generate a regular non-harmonic oscillation.

The SOTC is a new form of self-organization studied by Mahmoodi et al. (2017, 2018b), which establishes a significant difference between critical slowing down and temporal complexity. In the case of a conventional form of criticality, where the control parameter is finely tuned so as to generate phase transition (Beig et al., 2015) the two forms of complexity can be mistakenly judged to be equivalent. When a system at criticality is assigned a distribution different from the equilibrium distribution, the system is expected to relax back toward equilibrium with infinitely slow motion (critical slowing down). When the number of units is finite the mean field fluctuates around the mean value and the time interval between consecutive origin re-crossings, temporal complexity, may be as slow as critical slowing down (Beig et al., 2015).

In the case of SOTC the time necessary to evolve toward the condition of temporal criticality is finite (Mahmoodi et al., 2017, 2018b). In the special case studied by Bologna et al. (2010), another form of criticality generated by the fine tuning of the control parameter, but combining crucial events with periodicity, the mean field does not have regular oscillations: the intensity of these oscillations and their frequency are modulated in time generating the sausage-like structure of the second panel from the top of Figure 1. SOTC of a set of cooperating oscillators is expected to spontaneously lead to the same qualitative results. Subordination theory affords a simple way of mimicking this process of organization, introducing *ad hoc* the crucial events, which actually are the results of a spontaneous self-organization.

The processes of phase transitions are characterized by IPL PDFs with indices expressing the universality of criticality. The construction of renormalization group theory made it possible to determine without a detailed knowledge of the micro-interactions of the system, the scaling nature of phase transitions. In the case of the brain the micro-units, whose dynamics depart from the erratic behavior of independent units to collective behavior at criticality, are neurons. However, in spite of the frequent use of the term SOC these models rest on tuning a control parameter to a critical value that establishes global properties making the micro-dynamics unimportant in favor the macro-dynamics of criticality. If a neuron fires all the neurons linked to it makes a step ahead toward the firing level. Criticality is a condition generated by a suitable value of the control parameter that establishes a complex dynamics characterized by temporal complexity, namely, the crucial events defined in section 1. An interesting example of “Self-organized criticality” is given by Levina et al. (2009). They propose a very interesting model generating super-criticality and sub-criticality as well as criticality, a puzzling result because we expect that a process of self-organization may lead only to criticality. We make the conjecture that SOTC may realize this wide set of condition depending on how the process of self-organization is realized.

### 6.2. Future research work

The results of this paper suggest promising directions to establish homeodynamics as a form of genuinely spontaneous organization. SOTC (Mahmoodi et al., 2017) affords the prescription to turn a set of independent Poisson units, fluctuating between the values ξ = 1 and ξ = −1, into a self-organized system, thereby yielding, with a finite number of units, temporal complexity and crucial events. It is important to stress that, as shown by Mahmoodi et al. (2017), SOTC is a generator of complexity in line with the important concept of *intermediate asymptotics* of Barenblatt (Goldenfeld, 1992; Barenblatt, 1996). This is made evident in Figures 2, 9. The deviation of the scaling in both the short-time and long-time regions from the complex prediction of Equation (5) is not due to the numerical inaccuracy of DEA. Rather it is a surprising benefit of this technique of analysis that does not rely on the evaluation of the second moment of the PDF *p*(*x, l*) of Equation (8), but is a direct consequence of the general scaling behavior of the PDF. Not only is the PDF not forced to be Gaussian, its exact function form is not important.

These remarks lead us to conclude that the subordination theory used in this paper is an appropriate way to mimic the self-organization of units characterized by periodicity, as manifest in their spectra.

The comments we make in section 3 on the surprising agreement between the physical meaning of SOTC and the architectonic structures of Fingelkurts and Fingelkurts (2005) deserves further study. In our view, the interesting review paper (Fingelrkurts et al., 2012) provides directions on how to approach the challenging problem of cognition especially if it is supplemented by the contribution that emotion is expected to afford to it (Tuladhar et al., 2018), presumably through the proper action that meditation and emotion may exert on the non-crucial events.

## Ethics statement

We base our analysis on data derived from Matran-Fernandez and Polo (2017), which are available in physionet.org Goldberger et al. (2000). Matran-Fernandez and Polo (2017) has the following information. The study received the approval from the Ethics Committee of the University of Essex, and consent was obtained from all participants in written form prior to the beginning of the experiment. Recruitment of volunteers was performed via advertising through the University of Essex's mailing lists in February-March 2013. Only participants above 18 years old were considered for the experiment. Moreover, given the high presentation rates that are used in the RSVP protocol, participants were also screened for any personal or family history of epilepsy. No other exclusion criteria were used. Using these criteria, no participants were excluded from the experiment. All participants completed the experiment and were included in the analysis. No power analysis was performed to calculate sample size.

## Author contributions

All authors listed have made a substantial, direct and intellectual contribution to the work, and approved it for publication.

### Conflict of interest statement

The authors declare that the research was conducted in the absence of any commercial or financial relationships that could be construed as a potential conflict of interest.
